# Altered Inhibitory Synaptic Transmission and Changes in GABAergic Markers in the Hippocampus of Genetic and Environmental Animal Model of Autism

**DOI:** 10.1007/s11064-025-04590-w

**Published:** 2025-10-30

**Authors:** Bohumila Jurkovičová-Tarabová, Peter Vargovič, Denisa Mihalj, Tomáš Havránek, Jana Jakubíková, Kristóf László, Zuzana Bačová, Ján Bakoš

**Affiliations:** 1 Center of Biosciences, Institute of Molecular Physiology and Genetics, Slovak Academy of Sciences, Bratislava, Slovakia; 2 Biomedical Research Center, Institute of Experimental Endocrinology, Slovak Academy of Sciences, Dubravska cesta 9, 845 05 Bratislava, Slovakia; 3 Faculty of Medicine, Institute of Anatomy, Comenius University in Bratislava, Bratislava, Slovakia; 4 Biomedical Research Center, Cancer Research Institute, Slovak Academy of Sciences, Bratislava, Slovakia; 5 Institute of Physiology, Medical School, University of Pécs, Pecs, Hungary; 6 Center of Neuroscience, University of Pécs, Pecs, Hungary; 7 Faculty of Medicine, Institute of Physiology, Comenius University in Bratislava, Sasinkova 2, 813 72 Bratislava, Slovakia

**Keywords:** Autism spectrum disorder, Hippocampus, Valproate, *Shank3*, Glutamatergic neurons, GABAergic neurons

## Abstract

Changes in hippocampal neurons are known to play a critical role in social memory deficits associated with autism spectrum disorders (ASD). Although the theory of excitatory-inhibitory imbalance in autism pathogenesis is well established, early developmental alterations in the hippocampus remain insufficiently characterized. Alterations in gamma-aminobutyric acid (GABA)ergic neurons and their markers are thought to underlie synaptic changes in inhibitory circuits. Therefore, this study was designed to: (1) quantify glutamatergic and GABAergic neuron populations in the hippocampus; (2) characterize inhibitory postsynaptic currents (IPSCs) in primary hippocampal neurons; and (3) assess gene expression of selected GABAergic markers in two autism-like animal models, namely *Shank3*-deficient mice and prenatally valproate (VPA)-exposed rats. A reduced proportion of GABAergic neurons was observed in the hippocampus of both models. An increase in the number of glutamatergic neurons was found only in the hippocampus of prenatally VPA-exposed rats. This was accompanied by a decrease in IPSC frequency in primary hippocampal neurons from prenatally VPA-exposed rats, while no significant changes were found in *Shank3*-deficient mice. Altered temporal dynamics of inhibitory synaptic transmission were demonstrated in both models by a decreased cumulative probability of inter-event intervals for inhibitory currents. Furthermore, reduced gene expression levels of *Gabarap* and *Gabarapl1* were detected in *Shank3*-deficient mice, whereas decreased *Gat1* expression level was found in prenatally VPA-exposed rats at postnatal day 5. These findings strongly support the excitatory-inhibitory imbalance hypothesis in ASD. Thus, genetic or environmentally induced GABAergic changes in the hippocampus may underlie hippocampus-dependent social memory alterations in ASD.

**Supplementary Information:**

The online version contains supplementary material available at 10.1007/s11064-025-04590-w.

## Introduction

Autism spectrum disorder (ASD) is a widely studied condition characterized by neurodevelopmental symptoms that include delayed speech, challenges in social communication and interaction, and stereotyped, purposeless motor behaviors [1, 2]. At the cellular and molecular levels, alterations occur in several brain regions including the hippocampus during early developmental stages [3]. Recent studies using autism-like animal models have demonstrated the crucial role of the hippocampus in social memory deficits linked to ASD, along with altered synaptic plasticity [4–6]. Changes in synaptic plasticity may be linked to an imbalance between excitatory and inhibitory signals, both at the structural and electrophysiological levels.

Although the theory of excitatory-inhibitory imbalance in the etiology of ASD is relatively well-known [7], the specific mechanisms during the early stages of brain development remain insufficiently explored. A recent study found that an imbalance between excitatory and inhibitory neurotransmitters contributes to the development of ASD [8]. These authors revealed that alterations in glutamate-related genes are strongly linked to autism severity, with brain regions expressing higher levels of glutamate and gamma aminobutyric acid (GABA)-related genes showing significant differences in cortical thickness between autistic and neurotypical individuals. In support of this, our previous work has shown that SH3 and multiple ankyrin repeat domains 3 (*Shank3)*-deficient mice, a model for neurodevelopmental disorder with autistic symptomatology, exhibit an imbalance between excitatory and inhibitory neurotransmission, characterized by reduced GABAergic markers, and altered social behavior and anxiety [9]. This also suggests that autism-like conditions are linked to GABA neurotransmitter imbalance throughout development. Meta-analysis of magnetic resonance spectroscopy studies revealed reduced GABA levels in autistic brain regions, indicating disrupted excitation/inhibition balance [10]. Also, other studies indicate dysfunction in the GABAergic system in ASD [11, 12], though it is unclear if these changes originate in the hippocampus and emerge during early development.

To assess the GABAergic system in the hippocampus, a range of markers can be utilized, alongside measurements of the inhibitory properties of GABAergic neurons [13–15]. Key components of GABAergic neurotransmission include enzymes involved in GABA synthesis and degradation. The GABAergic system also comprises vesicular transporters (vGATs) and membrane transporters (GAT1, GAT3), which mediate GABA reuptake and synaptic inhibition [14, 16]. Additionally, members of the GABARAP protein family and scaffolding proteins, including Gephyrin, play crucial roles in receptor trafficking and anchoring of GABA receptors in postsynaptic membranes, contributing to the regulation and maintenance of inhibitory synaptic function [17].

Therefore, this study aimed to (1) analyse changes in the number of glutamatergic and GABAergic neurons in the hippocampus (2) examine the properties of inhibitory postsynaptic currents in primary hippocampal neurons and (3) evaluate gene expression of selected GABAergic markers in two autism-like animal models on postnatal day 5.

## Materials and Methods

### Chemicals and Reagents

In this study, the following chemicals were used: sodium valproate (VPA, P4543, Merck, Germany), Hank’s Balanced Salt Solution (HBSS, 14180-046, Thermo Fisher Scientific, Germany), further supplemented with 100 U/ml penicillin, 100 U/ml streptomycin (15140-122, Thermo Fisher Scientific, Germany) and 0.3 M Hepes (H6147, Sigma-Aldrich, Germany). Trypsin (15090-046, Thermo Fisher Scientific, Germany), DNAse I (11284932001, Roche), RPMI 1640 medium (R0883, Sigma-Aldrich, Germany) containing 10% fetal bovine serum (FBS, P40-37500, Pan Biotech, UK), flow cytometry staining buffer (SB, 00-4222-57, Thermo Fisher Scientific, Germany), Triton X-100 (T8787, Sigma-Aldrich, Germany), goat serum (NGS, G6767, Sigma-Aldrich, Germany) and 3% normal donkey serum (NDS, S30-M, Sigma-Aldrich, Germany). Poly-D-lysine hydrobromide (P6407, Sigma Aldrich, Germany).

Primary antibodies: glutamate decarboxylase 65/67 (GAD65/67) (1:500, rabbit anti-GAD65/67, ab11070; Abcam, UK), vesicular glutamate transporter protein 2 (VGLUT2) (1:500, chicken anti-VGLUT2, 135416, Synaptic System, Germany), for neuronal marker NeuN-AF488 (1:500, EPR12763, Abcam, Germany), the secondary antibodies for GAD: anti-rabbit Alexa Fluor™ Plus 405 (1:500, A48254 Thermo Fisher Scientific, Germany), for VGLUT2: anti-chicken Alexa Fluor 647 (1:500, A-21,449; Thermo Fisher Scientific, Germany).

Neuron-selective-growth medium containing Neurobasal A (10888022, Thermo Fisher Scientific, Germany), 100 U/ml penicillin; 100 U/ml streptomycin (15140-122, Thermo Fisher Scientific, Germany); 2 mM L-glutamine (25030-081, Thermo Fisher Scientific, Germany) enriched with 2% B27 supplement (I17504044, Invitrogen, USA) was used.

For electrophysiological measurements following reagents were used: extracellular solution contained (in millimolar): NaCl 140; KCl 3, CaCl2 2; MgCl2 1; HEPES 10; glucose 10; pH 7.4 (adjusted with NaOH) and intracellular solution contained (in millimolar): CsCl 135; NaCl 3, CaCl2 0.5; MgCl2 1, Na-ATP 4; Na-GTP 0.3; HEPES 10; EGTA 10; pH 7.4 (adjusted with CsOH). All chemicals used for patch-clamp recording solutions were purchased from Sigma Aldrich, Germany. Specific blockers N-ethyllidocaine (QX-314, Tocris Bioscience, UK), 6-cyano-7- nitroquinoxaline-2,3-dione (CNQX, Sigma Aldrich, Germany), D-2-amino-5-phosphonopentanoic acid (D-AP5, Sigma Aldrich, Germany) were used.

For qPCR analyses following reagents were used: TRIzol Reagent (15596026, Invitrogen, USA), High-Capacity cDNA Reverse Transcription Kit (4368814, Applied Biosystems, USA), Power SYBR^®^ Green PCR Master Mix (4367659, Applied Biosystems, USA).

### Animals

In these experiments we utilized two distinct animal models for ASD; *Shank3*^*−/−*^ mice (here also called *Shank3*- deficient mice) and valproic acid (VPA) autism rat model. *Shank3*-deficient mice served as a model for genetically based ASD, while rats prenatally exposed to valproate (VPA) represented an environmentally induced form of the disorder. Previous studies, including ours, have demonstrated that both models exhibit behavioral characteristics of autism-like behavior [18, 19]. All animals were kept on standard water supply and pelleted diet *at libitum*, group-housed (3–4 animal/cage) in temperature and light-controlled room (22 °C, 12 h light/12 h dark). All experimental procedures followed the ethical guidelines for animal experiments of the European Union Council (86/609/EEC) and were approved by the State Veterinary and Food Administration of the Slovak Republic and Hungary (5467-3/2023 − 220 and BA02/2000-82/2022 for rats; 6867/2024 − 220 for mice). Wild-type (WT) and homozygous knockout (*Shank3*^*−/−*^) mice were generated by mating of *Shank3B* heterozygous mice (B6.129-*Shank3*^tm2Gfng/J^) with PDZ depletion (exons 13 to 16) [13], obtained from the Jackson Laboratory (Stock No: 017688). Wild-type and *Shank3*^*−/−*^ mice genotype status was confirmed via polymerase chain reaction (PCR) using DNA extracted from liver, following the Jackson Laboratory protocol. The following primers were used - common Fw: GAGACTGATCAGCGCAGTTG; WT Rv: TGACATAATCGCTGGCAAAG; SHANK3^−/−^ Rv: GCTATACGAAGTTATGTCGACTAGG. The resulting PCR products were analyzed by gel electrophoresis. An autism-like rat model using prenatal VPA exposure was used, based on previous studies [20]. Gestating Wistar rats were purchased from the Charles River Laboratories (Germany). On embryonic day 12.5–13, pregnant dams received a single intraperitoneal injection of 450 mg/kg VPA or saline solution (*n* = 8/group).

In both experimental paradigms, *Shank3*-deficient mice and prenatal VPA exposure, tissue samples were collected either at postnatal day 0 for cell culture preparation or brains were dissected at postnatal day 5 for gene expression analyses.

### Isolation and Maintenance of Primary Hippocampal Cell Cultures

Hippocampi from P0 littermates’ animals (*n* = 6–8 for *Shank3*-deficient mice, (*n* = 10–12 prenatally VPA-treated rats, and their respective controls) were placed in ice-cold sterile HBSS. Hippocampi were individually collected under stereoscopic microscope and enzymatically dissociated for 20 min at 37 °C (HBSS, 0.1% Trypsin, 0.1 mg/ml DNAse I). To stop the enzymatic reaction medium was replaced by RPMI 1640 medium containing 10% FBS for 5 min at 37 °C (twice). Dissociation of the cells was achieved by gentle pipetting.

### Flow Cytometry

Single-cell suspension of hippocampal cells was prepared from the digested tissue used for preparation of primary neurons. Since there was a large loss of cells during the cell staining procedure involving numerous washing steps, samples were pooled from two animals to ensure sufficient material. Each sample was slowly passed through 1 mL and 200 µL automatic micropipettes for several times and filtered through 70 μm cell strainer (Falcon, BD, USA). After centrifugation at 300×g, 4 °C for 10 min., cells were fixed with 4% paraformaldehyde for 15 min at room temperature and two-times washed with flow cytometry SB. Cells were permeabilized with 0.1% Triton X-100 and non-specific antibody binding was blocked with 3% NGS and 3% NDS in SB for 30 min. For staining neurons, cell suspensions were incubated in SB containing 0.1% Triton X-100, 1% NGS/NDS and primary antibodies for GAD65/67 and VGLUT2 at 4 °C, overnight. Cells were two times washed with SB + 0.1% triton X-100 and 30 min incubated at 4 °C with fluorescence conjugated antibodies for neuronal marker NeuN-AF488, the secondary antibodies for GAD and VGLUT2. Cells were washed two times with SB + 0.1% triton X-100, resuspended in SB, and filtered again through a 70 μm cell strainer. Samples were measured using BD FACS Aria II SORP UV (BD Biosciences, USA). Collected data were analyzed in FlowJo (Tree Star, USA) and the representative gating is shown in the Supplementary data. Flow cytometry events were gated using forward and side scatter to distinguish cells from debris and following NeuN-positive signals (NeuN^+^) relevant to neuronal cells were gated for VGLUT2 and GAD65/67 detection. Threshold of specific fluorescence signal was set according to negative controls for VGLUT2 and GAD65/67 stained with absence of primary antibodies. For VGLUT2 we evaluated two neuronal population according to signal intensity, i.e. neurons with low (VGLUT2^low^) and high (VGLUT2^high^) fluorescence signal (see gating details in the Supplementary material).

### Measurement of Inhibitory Postsynaptic Currents (IPSCs)

Primary hippocampal cells were planted into 24-well plates with individual 12 mm round poly-D-lysin pre-coated cover slips and initially incubated for 3 h in RPMI 1640 medium containing 10% FBS under standard conditions (37 °C and 5% CO2). After pre-incubation RPMI medium was removed and exchanged for neuron-selective-growth medium for 5 days. Afterwards, 50% of the neuron-selective growth medium volume was exchanged every two days.

Recordings were performed in a whole-cell configuration of the voltage-clamp at -70 mV using a HEKA EPC10 amplifier (HEKA Electronics, Germany). Acquisition and analysis were performed using Patchmaster v90.2. The input resistance and capacity transients were compensated by up to 70% with built in circuits of the EPC 10 amplifier. Data were acquired at 10 kHz and filtered at 2.4 kHz. IPSCs were recorded in the primary culture of hippocampal neurons on DIV 10–14. Patch pipettes had a resistance ranging from 2.8 MΩ to 3.5 MΩ when filled with a intracellular solution (composition above). The osmolarity of the intracellular solution was approximately 300 mOsmol/L. During recordings, the pipette solution was supplemented with 5 mM N-ethyllidocaine in order to block action potentials in the patch-clamped neuron. Specific glutamate receptor blockers, 10 µM CNQX and 20 µM D-AP5 were applied to isolate the GABAergic postsynaptic currents. Recorded IPSCs were analyzed offline using automatic detection software Easy Electrophysiology (Easy Electrophysiology Ltd.,UK) and subsequently manually checked for accuracy. The detection threshold amplitude was set at 10 pA.

### Reverse Transcription Polymerase Chain Reaction Assays

Total RNA was isolated from the hippocampus using the phenol-chloroform method with TRIzol Reagent. The concentration and purity of the isolated RNA were determined by UV absorbance using a NanoDrop 2000 spectrophotometer (Thermo Fisher Scientific, USA). cDNA samples were generated using a High-Capacity cDNA Reverse Transcription Kit and stored at -20 °C until subsequent analysis. Reverse transcription quantitative real-time polymerase chain reaction (RT-qPCR) assays were performed using specific primers designed with NCBI Primer-BLAST (sequences listed in Table 1) and Power SYBR^®^ Green PCR Master Mix on a QuantStudio5 Real-Time PCR System (Applied Biosystems, USA). The relative expression levels of the selected genes were calculated using the 2^−ΔΔCt^ method, in which the threshold cycle (Ct) values were first normalized to the reference gene *Gapdh* in the same sample and then to the average Ct value of the control samples.

**Table 1:** List of primer sequences used in this study

Name	Primers	Gene Bank	References
*mAbat*	Fw: GGACTTCCGTCTTCATGAGTG Rv: ACCTCCACCTCTTCATACCT	NM_172961.3	[21]
*rAbat*	Fw: GAGGCCGTGCACTTTTTCTG Rv: CGCGTTTTGAGGCTGTTGAA	NM_031003.2	[22]
*mGabarap*	Fw: TTCTTGATCCGGAAGCGAAT Rv: CTGGTACAGCTGACCCATCG	NM_019749.4	[23]
*rGabarap*	Fw: CTTTCCCCTTGTTTACCCTCCAT Rv: CCCAATGTCAACCCCTTCCAT	NM_172036.4	[24]
*mGabarapl1*	Fw: CATCGTGGAGAAGGCTCCTA Rv: ATACAGCTGGCCCATGGTAG	NM_020590.4	[25]
*rGabarapl1*	Fw: CCTCCGACCTCACTGTTGG Rv: TGCCTCATTTCCCGTAGACAC	NM_001044294.1	[26]
*mGabarapl2*	Fw: TCACTGTGGCTCAGTTCATG Rv: TAGTTAGGCTGGACTGTGGG	NM_026693.5	[27]
*rGabarapl2*	Fw: TGGAACACAGATGCGTGGAA Rv: GTTAGGCTGGACTGTGGGAC	NM_022706.2	[28]
*mGat1*	Fw: TAACAACAACAGCCCATCCA Rv: GGAGTAACCCTGCTCCATGA	NM_178703.4	[29]
*rGat1*	Fw: TGCAAACACGTACGCACATAGAA Rv: AGATGCCTCAGCCACACCAC	NM_024371.3	[30]
*mGat3*	Fw: CTATGATGCCCCTCTCTCCAC Rv: CTGTCACAAGACTCTCCACG	NM_172890.3	[13]
*rGat3*	Fw: CGGTCACTGGAACAACAAGGTG Rv: AACACCACGTAAGGAATCAGGAATG	NM_024372.2	[31]
*mGapdh*	Fw: CGGTGCTGAGTATGTCGTGGA Rv: CTTTTGGCTCCACCCTTCAAG	NM_001289726.2	[32]
*rGapdh*	Fw: TGCACCACCAACTGCTTAG Rv: GGATGCAGGGATGATGTTC	NM_017008.4	[33]
*mGephyrin*	Fw: GACAGAGCAGTACGTGGAACTTCA Rv: GTCACCATCATAGCCGTCCAA	NM_145965.2	[34]
*rGephyrin*	Fw: ACCTCTGGGCATGCTCTCTA Rv: TGAAAGCATTCCTGAGATCC	NM_022865.4	[35]
*mVgat*	Fw: GGTGAAGTTCTACATCGACGTCAAG Rv: GTGTCCAGTTCATCATGCAGTGGA	NM_001421187.1	[36]
*rVgat*	Fw: GGGCTGGAACGTGACAAA Rv: GGAGGATGGCGTAGGGTAG	NM_031782.2	[37]

Forward (Fw), reverse (Rv); 4-aminobutyrate aminotransferase (*Abat*), GABA_A_R receptor-associated protein (*Gabarap*), GABA_A_R receptor-associated protein-like (*Gabarapl*), glyceraldehyde 3-phosphate dehydrogenase (*Gapdh*), GABA transporter (*Gat*), vesicular GABA transporter (*Vgat*)

### Statistical Analysis

Statistical analyses were performed using GraphPad Prism 10.2.3. Outliers in electrophysiological measurements were identified using the GraphPad ROUT method, which combines Robust regression and Outlier removal. Cleared data were subsequently tested for normality using Shapiro-Wilk test and a comparison of data for two groups was performed by Mann-Whitney non-parametric test. According to the results of the normality test, data for cumulative probability distributions were analysed with either Two-way ANOVA or Kruskal-Wallis test. A Student’s t-test with Welch´s correction was utilized to compare the data obtained from the flow cytometry and gene expression analysis. All results are expressed as mean ± SEM. The value of *p* < 0.05 was considered statistically significant.

## Results

### Hippocampal Cells Count Differences in *Shank3-*deficiency and Prenatal VPA Exposure

**Fig. 1:**
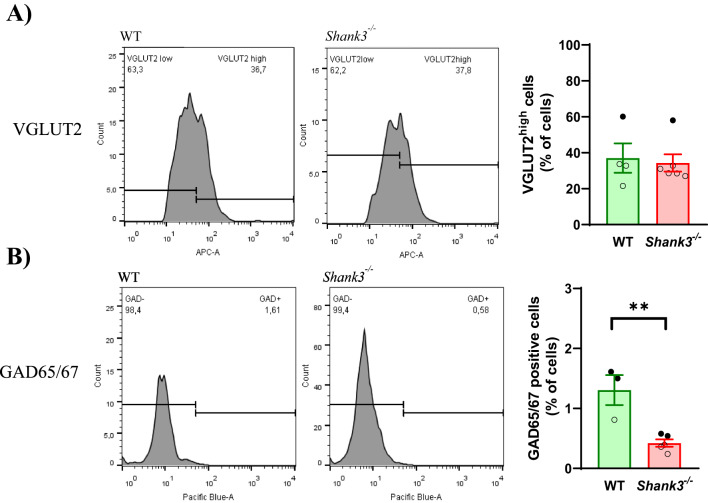
The proportion of glutamatergic and GABAergic neurons in the hippocampus isolated from *Shank3*-deficient mice. Flow cytometry histograms for **A** VGLUT2 stained neurons distinguished according to intensity of VGLUT2 signal to VGLUT2^low^ and VGLUT2^high^ populations. Comparison of the percentage of VGLUT2^high^ neurons from Wild type (WT, *n* = 3–4) and *Shank3* deficient mice (*Shank3*^*−/−*^ *n* = 5–6). **B** Histograms and comparison of the percentage of GAD65/67-positive neurons from WT and *Shank3* deficient mice. Values are presented as mean ± SEM. The p-values of the Student’s t-test are indicated. **p < 0.01

**Fig. 2:**
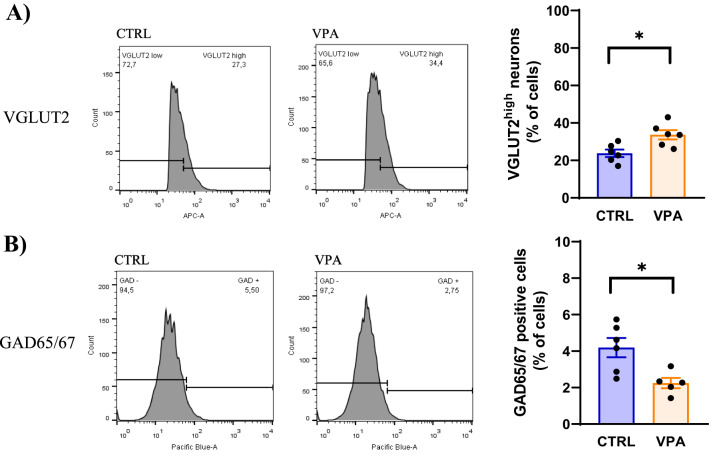
The proportion of glutamatergic and GABAergic neurons in the hippocampus isolated from rats prenatally exposed to valproic acid (VPA). Flow cytometry histograms for **A** VGLUT2 stained neurons distinguished according to intensity of VGLUT2 signal to VGLUT^low^ and VGLUT2^high^ populations. Comparison of the percentage of VGLUT2^high^ neurons from control rats (CTRL, *n* = 12 animals/6 samples) and VPA-exposed offspring (*n* = 10-12 animals/5-6 samples), each sample was pooled from two animals, **B** histograms and comparison of the percentage of GAD65/67-positive cells isolated from CTRL and VPA rats. Values are presented as mean ± SEM. The p-values of the Student’s t-test are indicated. *p < 0.05

Flow cytometry analysis (Fig. 1) of hippocampal cells isolated from WT and *Shank3*-deficient mice showed significantly reduced percentage of GAD65/67-positive cells in *Shank3*-deficient mice compared to WT mice (*p* < 0.01; t=4.409; df=6). No significant difference was found within populations of VGLUT2-positive cells. In the experiment of prenatal VPA exposure (Fig. 2), followed by the isolation of hippocampal cells from the offspring of control and VPA-exposed animals, the results revealed significant increase of neuronal population with high VGLUT2 content (VGLUT2^high^) in offspring of VPA-exposed rats compared to control rats (*p* < 0.05; t=3.112; df=9.571). Conversely, the GAD65/67-positive population showed a decrease in VPA-exposed than in control animals (*p* < 0.05; t=3.244; df=7.483).

### *Shank3*-Deficiency Alters Inhibitory Synaptic Transmission

**Fig. 3:**
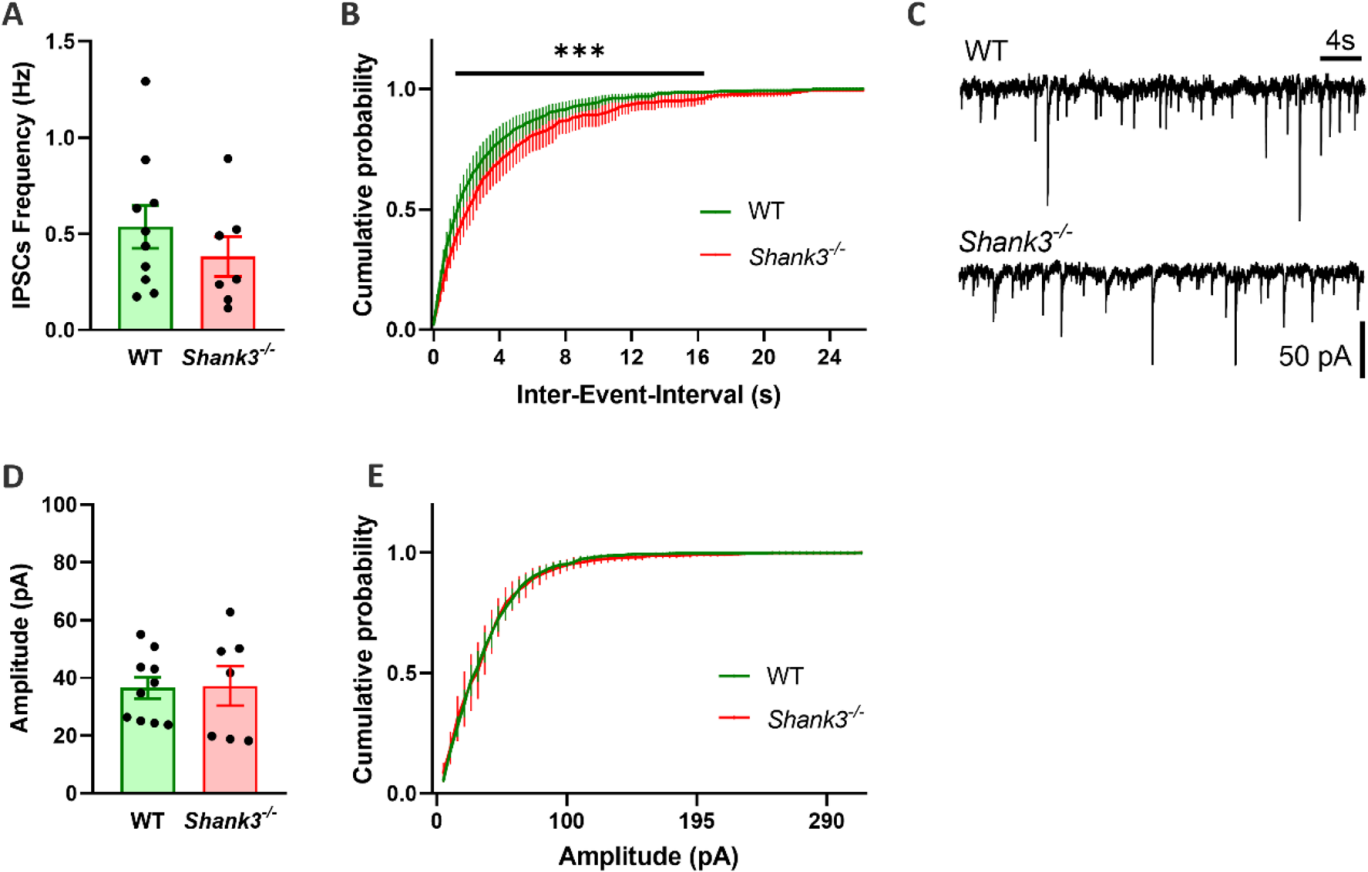
Inhibitory postsynaptic currents (IPSCs) in hippocampal neurons isolated from wild-type and *Shank3*-deficient mice. **A** Averaged IPSC frequencies recorded at -70 mV. **B** Cumulative probability distributions of IPSC frequency **C** Representative IPSC traces from WT and *Shank3*-deficient neurons. **D** Averaged IPSC amplitude in WT and *Shank3*-deficient mice neurons. **E** Cumulative probability distribution of IPSC amplitude. Values represent recordings of individual WT (*n* = 10) and *Shank3*-deficient (*n* = 7) neurons. Data are presented as mean ± SEM. Data in A and D were analysed by a Student’s t-test with Welch´s correction, data in B and E were analysed with Two-way ANOVA, significant differences indicated as ***p < 0.001

To assess the impact of *Shank3*-deficiency on inhibitory synaptic transmission IPSCs parameters were compared in WT and *Shank3*-deficient hippocampal neurons (Fig. 3). The mean IPSC frequency was slightly reduced in *Shank3*-deficient neurons relative to WT group, but this difference was not statistically significant. Similarly, no significant difference in IPSC amplitude distributions between the two groups was observed. However, the cumulative probability distributions of IPSC frequency showed a significant shift toward reduced event frequency (*p* < 0.001; F_(1, 1965)_ = 69.81), while amplitude remained unchanged in amplitude.

### Prenatal VPA Exposure Reduces Inhibitory Synaptic Event Frequency Without Affecting Amplitude

**Fig. 4:**
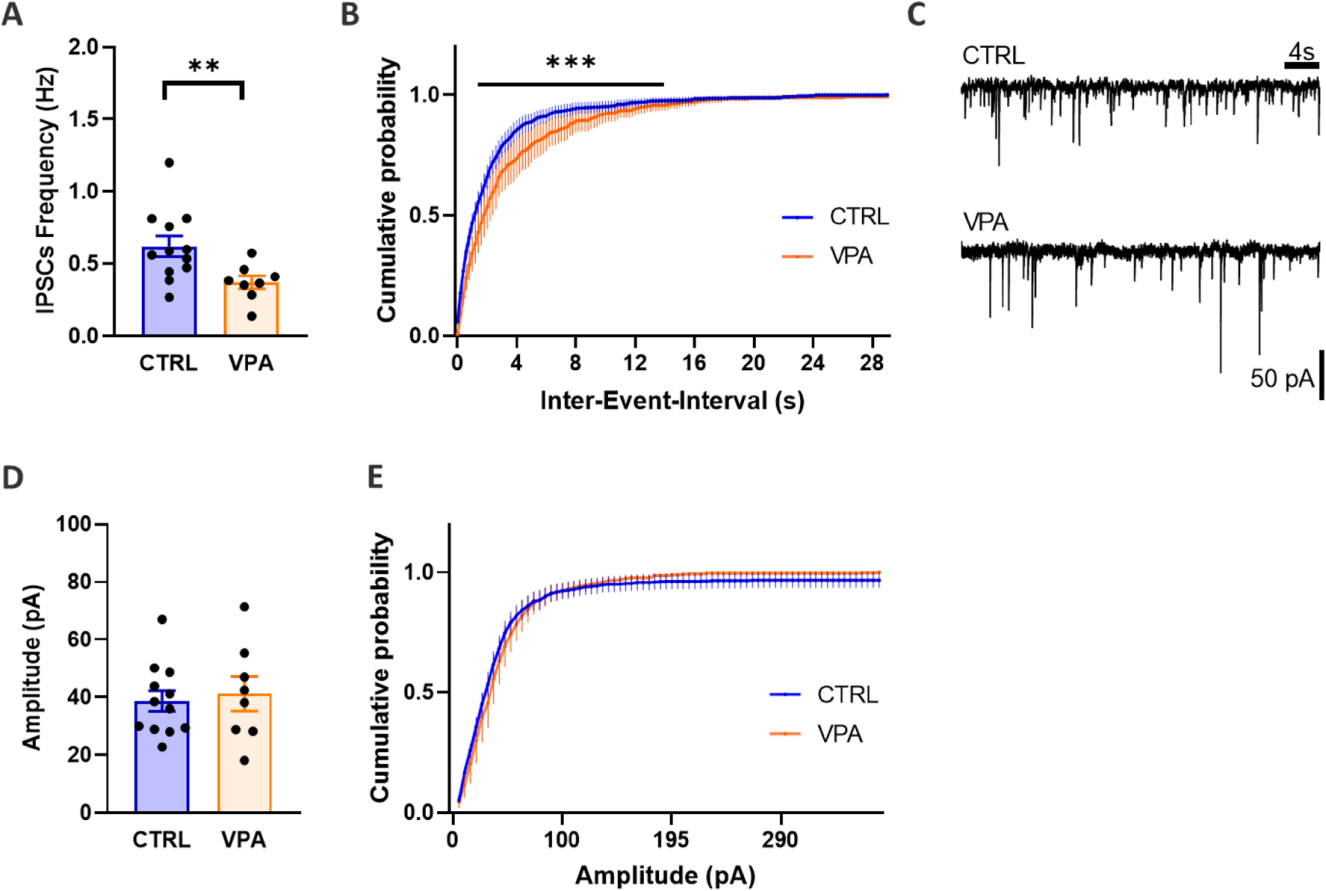
Inhibitory postsynaptic currents (IPSCs) in hippocampal neurons from control and valproic acid (VPA)- exposed rats. **A** Averaged IPSC frequencies recorded at -70 mV in neurons from control (CTRL) and VPA-exposed rats. **B** Cumulative probability distributions of IPSC frequency. **C** Representative IPSCs traces from CTRL and VPA-exposed rat neurons. **D** Averaged IPSC amplitude in neurons from CTRL and VPA- exposed rats. **E** Cumulative probability distributions of IPSC amplitude. Values represent recordings of individual CTRL (*n* = 12) and VPA (*n* = 8) neurons. Data are presented as mean ± SEM. Data in A and D were analysed by a Student’s t-test with Welch´s correction, data in B and E were analysed with Two-way ANOVA, significant differences indicated as: **p < 0.01, ***p < 0.001

The effects of prenatal VPA exposure on inhibitory synaptic activity were assessed by recording spontaneous IPSCs in hippocampal neurons (Fig. 4). VPA-exposed neurons exhibited a significantly reduced frequency of inhibitory events. A statistically significant reduction in IPSC frequency was observed in the VPA compared to CTRL group (*p* < 0.01; t = 2.952; df = 17.21). The cumulative probability distribution of IPSC frequency confirmed this decrease (*p* < 0.001; F_(1, 2628)_ = 108.7). In contrast, IPSC amplitudes did not differ significantly between the groups, with similar median values and cumulative amplitude distribution. These results suggest that VPA exposure impairs the frequency of inhibitory events, thus primarily disrupting presynaptic inhibitory circuitry, but postsynaptic receptor function is not affected.

### Unchanged IPSC Kinetics with Increased Variability in *Shank3-*Deficiency and Prenatal VPA Exposure

The kinetics of IPSCs in hippocampal neurons were evaluated across both models, including the rise time, decay time and event half-width constants. No significant changes in these parameters were observed across all groups (WT, *Shank3*^*−/−*^, control, and VPA-exposed). Rise times remained stable, while half-width and decay times showed greater variability, particularly in the *Shank3*-deficient and prenatally VPA-exposed groups compared to controls. These findings suggest heterogeneity in inhibitory synaptic maturation during neurodevelopment.

### Reduced GABAergic Marker Expression Is Associated with both *Shank3-*Deficiency and Prenatal VPA Exposure

The gene expression levels of selected presynaptic and postsynaptic proteins were analyzed in the hippocampus of 5-day-old pups in both animal models (Table 2). Gene expression analysis showed a significant reduction in the mRNA levels of GABA_A_ receptor-associated protein (*Gabarap*; *p* < 0.01; t = 3.68; df = 9) in the hippocampus of *Shank3*-deficient mice compared to WT mice. *Gabarap*-like protein 1 (*Gabarapl1*) expression levels showed a trend toward reduction in the hippocampus of *Shank3*-deficient mice, but these changes were not statistically significant (*p* = 0.056; t = 2.20; df = 9). Prenatal VPA exposure resulted in a statistically significant decrease in the gene expression of GABA transporter 1 (*Gat1*; *p* < 0.05; t = 2.64; df = 8) in the hippocampus compared to the CTRL rats. The gene expression analysis of other GABA markers in both autism-like models did not yield statistically significant changes.

**Table 2:** Transcript levels of selected GABAergic presynaptic and postsynaptic markers in the hippocampus of two autism-like models at postnatal day 5

Gene	Shank3^−/−^ mice		VPA-treated rats	
	WT	*Shank3* ^*−/−*^	CTRL	VPA
*Abat*	1.04 ± 0.18	1.10 ± 0.19	1.02 ± 0.14	0.76 ± 0.08
*Vgat*	1.10 ± 0.31	1.14 ± 0.21	1.00 ± 0.08	1.10 ± 0.04
*Gat1*	0.99 ± 0.13	1.27 ± 0.58	1.03 ± 0.18	0.55 ± 0.05*
*Gat3*	1.00 ± 0.07	0.81 ± 0.12	1.03 ± 0.17	0.89 ± 0.07
*Gephyrin*	1.01 ± 0.08	0.75 ± 0.18	1.09 ± 0.27	0.86 ± 0.14
*Gabarap*	1.00 ± 0.05	0.72 ± 0.05**	1.00 ± 0.10	1.11 ± 0.15
*Gabarapl1*	1.00 ± 0.06	0.76 ± 0.09	1.02 ± 0.13	0.84 ± 0.17
*Gabarapl2*	1.00 ± 0.06	0.85 ± 0.08	1.02 ± 0.16	0.90 ± 0.14

Values represent relative mRNA levels normalized to *Gapdh* transcript and compared to the wild-type (WT) or control (CTRL) group calculated by the 2^−ΔΔCt^. Table shows the mean ± SEM (*n* = 3–6/genotype; *n* = 5–6/group). Statistical significance between values was determined using the two-tailed student’s t-test (*p < 0.05; **p < 0.01). 4-aminobutyrate aminotransferase (*Abat*), GABA_A_ receptor-associated protein (*Gabarap*), GABA_A_ receptor-associated protein-like (*Gabarapl*), glyceraldehyde 3-phosphate dehydrogenase (*Gapdh*), GABA transporter (*Gat*), valproate exposure (VPA), vesicular GABA transporter (*Vgat*)

## Discussion

In the present study, we found a reduced proportion of GABAergic neurons in the hippocampus isolated from both autism-like models. This finding corresponds to the decreased IPSC frequency observed in primary hippocampal neurons from prenatal VPA-exposed rats, although no such significant differences were observed in hippocampal neurons from *Shank3*-deficient mice. Both models showed a decreased cumulative probability of inter-event intervals for inhibitory currents, indicating altered temporal dynamics of inhibitory synaptic transmission within the hippocampal network. Furthermore, *Shank3*-deficient mice showed decreased gene expression of *Gabarap* and *Gabarapl1*, while prenatally VPA-exposed rats showed only reduced *Gat1* expression.

The reduction in the number of GABAergic neurons in the hippocampus of both autism-like animal models corresponds to our previous findings demonstrating reduced GAD65/67 expressions in the brains of *Shank3*-deficient mouse pups and adults [9]. Expression of GAD enzyme is commonly used as a proxy to estimate GABAergic neurons in the hippocampus in various studies [14]. Similarly, a recent study indicated that prenatal VPA exposure leads to decreased expression of GAD67 in the hippocampus during early development [38]. Additionally, single-cell transcriptomics has revealed a decrease in GABAergic neurons and reduced GABA levels in cortical areas of the brain in a different model exhibiting autistic-like behaviors [39]. Our results also demonstrated an increase in the number of neurons with high level of VGLUT2 (VGLUT2^high^ population), but only in the hippocampus isolated from prenatal VPA-exposed rats. Previous studies reported increased glutamate and glutamine levels in the hippocampus of prenatally VPA-exposed rats at postnatal day 14 [40] and altered neuronal activity and reduced dendritic complexity in hippocampal neurons of prenatally VPA-exposed rats [41]. Together, our findings and previous studies suggest an excitatory-inhibitory imbalance in the hippocampus during the perinatal period, with potential functional implications.

In this study, specifically, prenatally VPA-exposed rats showed decreased IPSC frequency in hippocampal neurons, while Shank3-*deficient* mice did not exhibit this alteration. This is consistent with previous studies reporting no changes in miniature inhibitory postsynaptic currents in CA1 pyramidal neurons in *Shank3*-deficient mice [13, 42]. Conversely, some studies, though not directly investigating hippocampal neurons, have reported a decreased frequency of spontaneous IPSCs in SHANK3 knockdown human neurons relative to control neurons [43]. In the present study, both models used exhibited altered inhibitory synaptic transmission timing in the hippocampus. Moreover, one study reported a significantly lower frequency of miniature inhibitory postsynaptic currents, which reflect IPSPs, across multiple developmental stages in CNTNAP2-knockdown neurons, a model commonly used to study autism pathogenesis [44]. These authors also found that the amplitude of IPSCs in CNTNAP2-knockdown neurons was significantly smaller than that of control neurons. These findings, together with changes in GABAergic neuron numbers, indicate significant alterations in inhibitory neurons that likely contribute to autistic symptoms, during early stages of brain development, particularly in the hippocampus. Although it is important to note that our study did not assess behavioral symptoms, we primarily focused on molecular changes in the hippocampus shortly after birth. We observed decreased gene expression of *Gabarap* and *Gabarapl1* in *Shank3*-deficient mice at postnatal day 5, which may relate to altered dynamics of inhibitory synaptic transmission in the hippocampus. This aligns with recent findings that GABARAP and GABARAPL1 regulate GABA(A) receptor trafficking and synaptic localization, and their disruption impairs GABAergic currents [45]. Although conclusions should be made cautiously, as we did not observe similar findings in prenatally VPA-exposed rats, which showed reduced *Gat1* expression. Under physiological conditions, GAT1 expression increases postnatally with inhibitory circuit maturation, facilitating GABA reuptake and synaptic inhibition in rodent animal models [46, 47]. A different study using postmortem human tissues reported a reduction in inhibitory synapses across all cortical layers of the prefrontal cortex in ASD brains compared to controls [48]. Thus, changes in IPSCs, the timing of inhibitory currents, and lower levels of GABAergic markers in the hippocampus clearly indicate alterations in inhibitory circuits in the two animal models used in this study.

It is important to note that early in postnatal development, GABA can have depolarizing or even excitatory effects due to higher intracellular chloride concentrations [49]. Some studies show that in hippocampal subregions, GABAergic input to apical dendrites stays depolarizing after it becomes inhibitory in the soma and basal dendrites [50]. It has also been proposed that a delay in the shift from excitatory to inhibitory GABAergic signaling may play a role in the etiology of ASD [51–53]. Hippocampal inhibitory effects of GABA appear by postnatal day 7 in vivo [54] and at DIV10 in vitro in rodents [55]. We measured currents at DIV10-14, therefore we assume GABAergic transmission was already inhibitory at that time, despite known regional heterogeneity in developing interneurons. An important limitation of our study is the lack of sex-based analysis, as sex was not distinguished during primary neuron isolation, and GABAergic marker analysis was performed only on tissues from males. Nevertheless, it should be noted that ASD is approximately four times more prevalent in males than females especially in early development [56], making the analysis of hippocampal tissue from male animals in both models highly relevant.

In summary, although inhibitory currents and GABAergic marker expression differ in both autism-like animal models, the decreased frequency of inhibitory synaptic events likely reflects reduced synaptic activity or fewer functional inhibitory synapses, consistent with the observed reduction in GABAergic neuron numbers shortly after birth. These findings strongly support the theory of excitatory-inhibitory imbalance in ASD, whether driven by genetic mutations or environmental factors, with alterations emerging early, before behavioral symptoms develop.

## Supplementary Information

Below is the link to the electronic supplementary material.

Supplementary Material 1

## Data Availability

The data that support the findings of this study are available from the corresponding author upon reasonable request.
