# Supplementary material for: Altered Inhibitory Synaptic Transmission and Changes in GABAergic Markers in the Hippocampus of Genetic and Environmental Animal Model of Autism
Source: Neurochem Res. 2025 Oct 30;50(6):340. doi: 10.1007/s11064-025-04590-w (PMC12575502; doi:10.1007/s11064-025-04590-w)
Supplement: Supplementary file 1 — Supplementary Material 1 [file 11064_2025_4590_MOESM1_ESM.docx]

**Supplementary data**


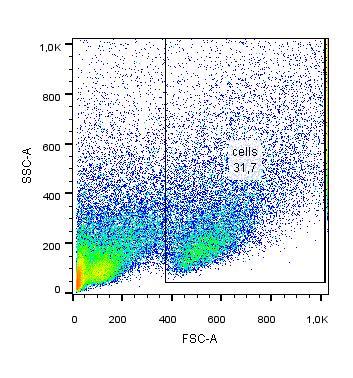


**A)** All events


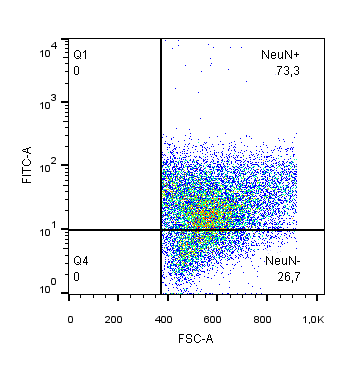


**B)** NeuN – positive cells

**C)** VGLUT2 positive neurons

**D)** VGLUT2 intensity

**E)** GAD65/67 - positive neurons


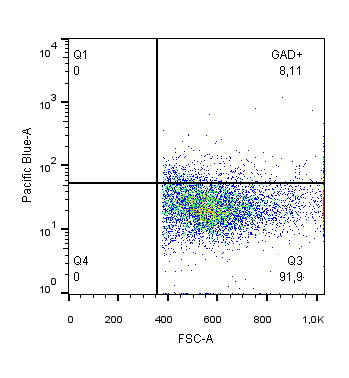

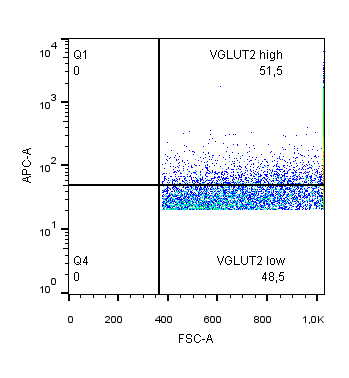

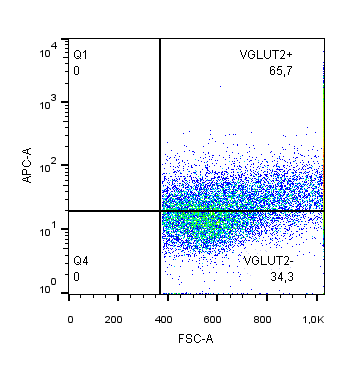


**Supplementary Figure 1. Gating strategy for flow cytometry of hippocampal neurons.** Flow cytometry plots showing (A) all events with gating of total cells population, (B) NeuN - positive cells (NeuN^+^) - neurons, C) all VGLUT2-positive neurons (VGLUT2^+^), D) VGLUT2^high^ and VGLUT2^low^ populations distinguished on the base of VGLUT2 signal intensity, E) percentage of GAD65/67-positive (GAD^+^) neurons gated from VGLUT2-negative (VGLUT2^-^) population.
